# GPR40 agonist ameliorates liver X receptor-induced lipid accumulation in liver by activating AMPK pathway

**DOI:** 10.1038/srep25237

**Published:** 2016-04-28

**Authors:** Meng Li, Xiangyu Meng, Jie Xu, Xiuqing Huang, Hongxia Li, Guoping Li, Shu Wang, Yong Man, Weiqing Tang, Jian Li

**Affiliations:** 1 Peking University Fifth School of Clinical Medicine (Beijing Hospital), Beijing, China; 2 The Key Laboratory of Geriatrics, Beijing Institute of Geriatrics & Beijing Hospital, Ministry of Health, Beijing, China

## Abstract

Hepatic steatosis is strongly linked to insulin resistance and type 2 diabetes. GPR40 is a G protein-coupled receptor mediating free fatty acid-induced insulin secretion and thus plays a beneficial role in the improvement of diabetes. However, the impact of GPR40 agonist on hepatic steatosis still remains to be elucidated. In the present study, we found that activation of GPR40 by its agonist GW9508 attenuated Liver X receptor (LXR)-induced hepatic lipid accumulation. Activation of LXR in the livers of C57BL/6 mice fed a high-cholesterol diet and in HepG2 cells stimulated by chemical agonist caused increased expression of its target lipogenic genes and subsequent lipid accumulation. All these effects of LXR were dramatically downregulated after GW9508 supplementation. Moreover, GPR40 activation was accompanied by upregulation of AMPK pathway, whereas the inhibitive effect of GPR40 on the lipogenic gene expression was largely abrogated by AMPK knockdown. Taken together, our results demonstrated that GW9508 exerts a beneficial effect to ameliorate LXR-induced hepatic steatosis through regulation of AMPK signaling pathway.

The liver is the main organ responsible for the lipid and glucose homeostasis, as well as energy metabolism in the whole body. Excess hepatic lipid deposition mainly induced by nutrition factors and genetic defects is the original cause of steatohepatitis, insulin resistance and many other liver injuries^1^,^2^. To control the onset and progress of these metabolic syndromes, it is necessary to understand the underlying mechanism of regulation of lipid accumulation in the liver. Considerable evidence indicates that liver X receptor (LXR) functions as a sterol sensor that protects cells from cholesterol overload^3^,^4^. There are two LXRs identified, LXRα and LXRβ. LXRα predominates in the adipose tissue, liver and macrophages, while LXRβ is expressed ubiquitously^5^. LXR responds to elevated cholesterol levels by regulating the expression of genes involved in stimulation of reverse cholesterol transport (ABCA1, ABCG1 and ApoE), cholesterol secretion (ABCG5 and ABCG8), cholesterol catabolism (CYP7A1), and in inhibition of cholesterol synthesis (SREBP2) *de novo*^6^. However, in addition to modulating cholesterol metabolism, LXR also regulates the hepatic fatty acid biosynthesis pathway by activating sterol regulatory element binding protein (SREBP)-1c^7^, which is the master regulator of hepatic lipogenesis^8^. Mice treated with synthetic LXR agonists experienced elevated triglyceride level in the liver and transiently in the plasma via upregulation of SREBP-1c and induction of fatty acid synthase (FAS), acyl-CoA carboxylase (ACC), and stearyl-CoA desaturase-1 (SCD1)^9^.

G protein-coupled receptors (GPCRs) are important signaling molecules that regulate a myriad of cellular physiological processes. Five free fatty acid (FFA) receptors (GPR40, GPR41, GPR43, GPR84, GPR120) of GPCR superfamily have been discovered^10^,^11^,^12^,^13^. GPR40, also referred to as FFAR1, couples with a G-protein α-subunit of Gq family to function as a receptor for a range of medium- to long-chain saturated and unsaturated fatty acids^14^. GPR40 was firstly detected in pancreatic β-cells and it increased Gq protein-mediated phospholipase C (PLC) activity and intracellular calcium levels in pancreatic islets and thus, was responsible for glucose-dependent stimulation of insulin secretion by fatty acids^11^. Recent studies have validated that GPR40 is also expressed in hepatocytes and exerts insulin sensitizing effect^15^,^16^. In addition, Ou *at el*.^17^ also found that activation of GPR40 exerted a beneficial effect to improve high fat diet-induced hepatic steatosis in C57BL/6 mice. Considering the important role of LXR in lipid metabolism, it’s of great interest for us to explore the effect of GPR40 on LXR activation-induced hepatic steatosis, which is still largely obscure.

Jung *et al*. showed that LXR agonist T0901317-induced hepatic steatosis was ameliorated by an ω-3 fatty acid (FA) diet, and this was accompanied by blunted upregulation of SREBP1-c and FAS expression caused by LXR activation^18^. However, ω-3 FA has also been reported to be an endogenous agonist of GPR40^19^. Based on these facts, we hypothesized that GPR40 might play an important role in LXR activation-stimulated lipid accumulation in liver. To directly test this hypothesis, we stimulated GPR40 with synthetic GPR40 agonist GW9508 in both C57BL/6 mice and HepG2 cells and examined whether activation of GPR40 affected high cholesterol diet- and synthetic LXR agonist T0901317-induced hepatic lipogenic pathway responses in mice and HepG2 cells respectively. Consistent with our hypothesis, GW9508 treatment markedly ameliorated lipid accumulation in the liver of C57BL/6 mice and in HepG2 cells induced by LXR activation and this effect was associated with stimulation of AMP-activated protein kinase (AMPK) signaling pathway, followed by decreased LXR-induced expression of FAS, ACC and SCD1.

## Results

### Administration of GW9508 prevents high cholesterol diet-induced hepatic lipid accumulation in C57BL/6 mice

Hepatic LXRα has been known to facilitate regulation of hepatic response to cholesterol challenge when mice were fed a high cholesterol diet^20^. To investigate whether activation of GPR40 relieved LXR activation-induced lipid accumulation in the liver of mice, C57BL/6 mice were fed a high-cholesterol diet (HCD) and treated daily with either vehicle or GW9508 at 100 mg/kg body weight by gavage for 3 days. After being fed HCD for 3 days, both vehicle- and GW9508-treated mice displayed significantly higher levels of serum total cholesterol (TC), free cholesterol (FC) and cholesterol ester (CE) than control mice, while the levels of serum triglyceride (TG), phospholipids (PL) and free fatty acid did not differ among the groups. Additionally, there were no changes in both body weight and food intake (Table 1).

**Figure 1:**
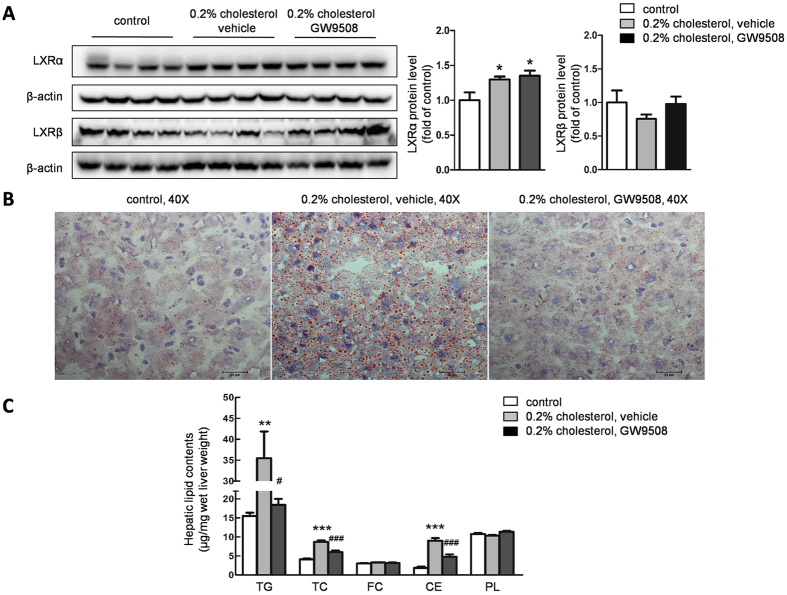
Intragastric administration of GW9508 ameliorates high cholesterol diet (HCD) - induced hepatic lipid accumulation in C57BL/6 mice. C57BL/6 mice were fed the 0.2% cholesterol diet for 3 days by gavage daily with either vehicle or GW9508 (100 mg/kg BW/day). After a 4 h fast, mice were sacrificed. Livers were collected from mice fed a control diet, a HCD supplemented with vehicle, and a HCD supplemented with GW9508. (**A**) LXRα and LXRβ expression was detected by western blot, data are presented as the mean ± SEM, *p < 0.05, versus control diet-fed mice. (**B**) Representative oil red O staining of liver cryosections, bar = 25 μm. (**C**) Hepatic lipid contents, data are presented as the mean ± SEM from 6–7 animals of each group. **p < 0.01, ***p < 0.001, versus control diet-fed mice; ^#^p < 0.05, ^###^p < 0.001, versus HCD-fed plus vehicle mice.

To verify that high serum cholesterol could affect hepatic LXR activity in mice, we detected the expression of hepatic LXRs by western blot. After 3-day HCD feeding, not only did the serum cholesterol concentration increase, but also the expression of hepatic LXRα was significantly upregulated in both vehicle- and GW9508-treated mice compared with control mice, whereas the expression of LXRβ was not affected (Fig. 1A). These data indicated that LXRα, the dominant isoform in liver, could be successfully activated by high cholesterol diet in mice.

Activated hepatic LXR could activate the genes involved in lipogenesis and led to hepatic steatosis. As we expected, oil red O staining of sections from livers in HCD-fed mice treated with vehicle revealed that hepatocytes were filled with small size lipid droplets. Strikingly, LXR activation-induced lipid accumulation in livers were largely absent in the GW9508 treatment group (Fig. 1B). Consistent with hepatic lipid accumulation, the contents of hepatic total cholesterol, cholesterol ester and triglyceride were 2.1-, 4.5- and 2.3-fold increased, respectively, in HCD fed-mice versus control mice. However, GW9508 administration largely decreased hepatic total cholesterol, cholesterol ester, and triglyceride levels of corresponding HCD group. There was no difference observed between the contents of hepatic free cholesterol and phospholipids among 3 groups (Fig. 1C).

### Administration of GW9508 suppresses LXR activation-induced upregulation of hepatic lipogenic gene expression and activates AMPK-ACC signaling pathway

**Figure 2:**
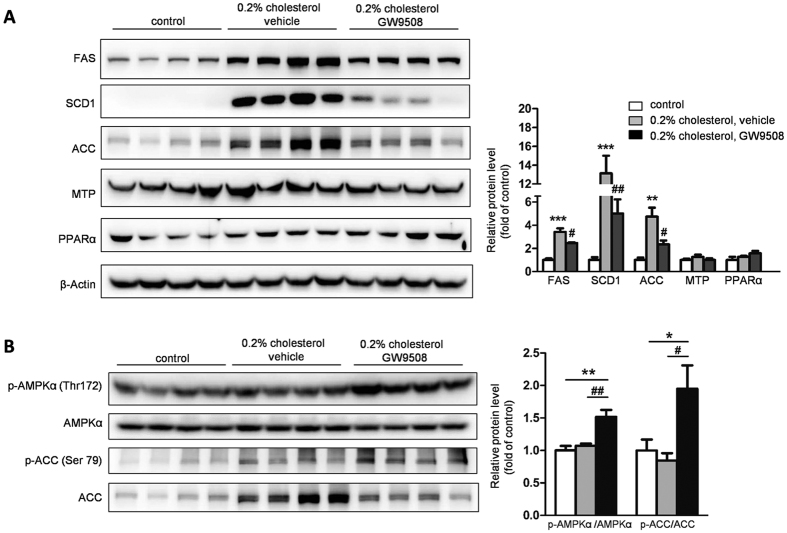
Administration of GW9508 suppresses LXR activation-induced upregulation of hepatic lipogenic gene expression and activates AMPK-ACC signal pathway in C57BL/6 mice. Western blot analysis of protein expression and corresponding densitometric quantification of FAS, SCD1, ACC, MTP, PPARα (**A**) and phosphorylation of AMPKα (Thr172) and ACC (Ser79) (**B**) were performed. The data are presented as the mean ± SEM. *p < 0.05, **p < 0.01, ***p < 0.001, versus control diet-fed mice; ^#^p < 0.05, ^##^p < 0.01, versus HCD-fed plus vehicle mice.

To study how GW9508 attenuated LXR activation-induced hepatic lipid accumulation, the expression of genes involved in hepatic triglyceride metabolism was detected using western blot. In mice challenged with 3-day HCD and vehicle, the expression of genes involved in fatty acid synthesis, such as FAS, SCD1, and ACC, was dramatically upregulated 3.4-, 13.1- and 4.7- folds, respectively, compared with control mice. However, the expression levels of microsomal triglyceride transfer protein (MTP) and peroxisome proliferator-activated receptors-alpha (PPARα) involved in very low density lipoprotein (VLDL) formation and triglyceride oxidation, respectively, remained unchanged. As expected, administration of GW9508 to HCD-fed mice caused 28%, 62% and 50% decreases in the expression of FAS, SCD1, and ACC, respectively, compared with HCD-fed plus vehicle-treated mice (Fig. 2A). Together, these results demonstrated that the attenuation of hepatic lipid accumulation in GW9508-treated mice was coupled with reduced hepatic lipogenesis, rather than triglyceride oxidation, VLDL formation, or secretion.

It’s showed that phosphorylation on Thr172 residue of AMPKα subunit may downregulate expression of genes involved in lipogenesis^21^. In some cell types, AMPK Thr172 can be phosphorylated by the Ca^2+^/calmodulin-dependent protein kinase CaMKKβ^22^. Moreover, GPR40 is a Gαq/11 coupled receptor, which results in activation of phospholipase C^23^ and subsequent increase in the intracellular Ca^2+^ concentration^24^. Based on these evidences, we speculated that GPR40 might induce the activation of AMPK and then prevent LXR-induced lipid accumulation in the livers of C57BL/6 mice. To examine this possibility, we analyzed the AMPK activity by measuring Thr172 phosphorylation of AMPKα, and Ser79 phosphorylation of ACC, a downstream target of activated AMPK. We found that HCD feeding in combination with vehicle treatment did not affect phosphorylation of AMPK and ACC in mice livers. However, HCD feeding in combination with GW9508 treatment significantly enhanced the phosphorylation of AMPK and ACC (Fig. 2B). Activation of AMPK led to the phosphorylation and concomitant activity inhibition of ACC, the rate-limiting enzyme controlling fatty acid synthesis^25^, suggesting one possible mechanism for lipid-lowering effect of GW9508 in liver.

### Activation of GPR40 by GW9508 attenuates LXR activation-induced triglyceride accumulation in HepG2 cells

**Figure 3:**
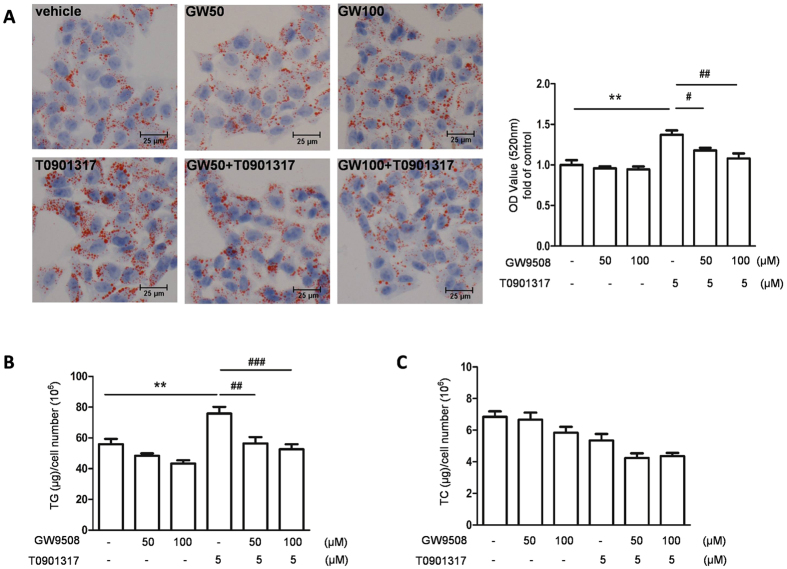
GW9508 attenuates LXR activation-induced triglyceride accumulation in HepG2 cells. HepG2 cells were pretreated with either 50 or 100 μM GW9508 for 1 h and then treated with 5 μM T0901317 for 48 h. (**A**) Oil red O staining showed lipid droplets, bar = 25 μm. The data was representative of three independent experiments. Oil red O was then extracted by isopropanol and quantified with spectrophotometry at 520 nm. Cellular total triglyceride (**B**) and cholesterol (**C**) were measured. Data are expressed as means ± SEM and obtained from 3 individual experiments. **p < 0.01, compared with the control group; ^#^p < 0.05, ^##^p < 0.01, ^###^p < 0.001, compared with T0901317-treated group.

To further explore the mechanism of GPR40 in the regulation of LXR-induced hepatic lipogenesis, we developed an *in vitro* model of hepatic steatosis by exposing HepG2 cells to 5 μM of T0901317, a synthetic agonist for LXR, and then challenged this model by stimulating HepG2 cells with either 50 or 100 μM of GW9508 for 1 h prior to T0901317 treatment. Oil red O staining indicated that T0901317 treatment induced lipid accumulation in HepG2 cells, while T0901317-induced lipid accumulation was largely reversed to normal level when together incubated with GW9508 (Fig. 3A). To further verify the function of GW9508, cellar contents of triglyceride and cholesterol were also measured. Consistent with oil Red O staining, triglyceride content after treated with LXR agonist T0901317 was higher than that in vehicle-treated cells, whereas GW9508 pretreatment converted the increased triglyceride content induced by T0901317 to normal level (Fig. 3B). Cholesterol content was not affected in the presence of GW9508 or T0901317 (Fig. 3C).

**Figure 4:**
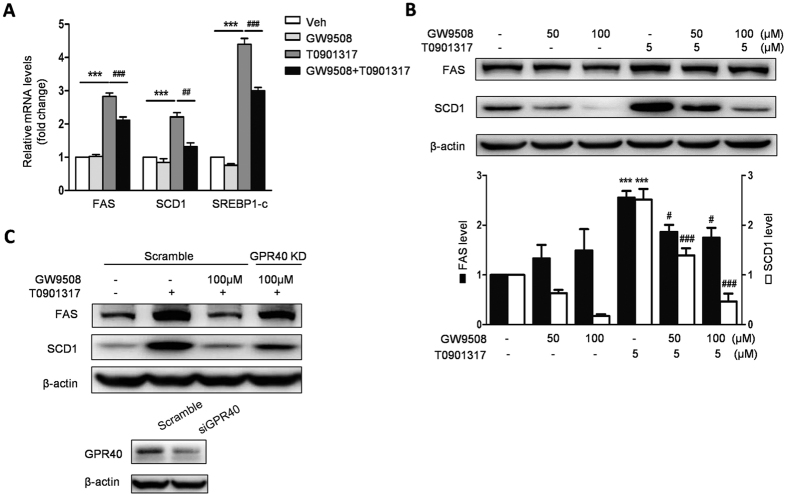
GW9508 ameliorates LXR activation-induced triglyceride accumulation through activation of GPR40. HepG2 cells were pretreated with either 50 or 100 μM GW9508 for 1 h and then treated with 5 μM T0901317 for 24 h. (**A**) Measurement of mRNA levels of lipogenic genes including SREBP1c, FAS, and SCD1 by real-time PCR. (**B**) Western blot analysis of FAS and SCD1 protein levels. Data are expressed as means ± SEM and obtained from 3 individual experiments. ***p < 0.001, compared with the control group; ^#^p < 0.05, ^##^p < 0.01, ^###^p < 0.001, compared with T0901317-treated group. (**C**) Protein expression of FAS and SCD1 when GPR40 was knocked down. HepG2 cells were transfected with siRNA of GPR40 or scrambled siRNA for 24 h and pretreated with 100 μM GW9508 for 1 h before 5 μM T0901317 treatment for 24 h.

To further assess that GW9508 reduced LXR activation-induced triglyceride accumulation in HepG2 cells through inhibiting expression of genes involved in lipogenesis, we measured the expression of SREBP1c, FAS, and SCD1 by q-PCR and western blot in the HepG2 cells treated with T0901317 and GW9508. Compared with control cells, T0901317 treatment caused 4.4-, 2.8- and 2.2- fold increase in mRNA levels for SREBP1c, FAS, and SCD1, respectively, and 2.6- and 2.5- fold increase in protein levels for FAS and SCD1, respectively. However, GW9508 pretreatment led to reduced T0901317-induced expression of SREBP1c, FAS and SCD1 (Fig. 4A,B). In addition, the inhibitory effect of GW9508 on the protein expression of lipogenic genes FAS and SCD1 were largely diminished by GPR40 knockdown (Fig. 4C). Collectively, GW9508 activated GPR40 and attenuated triglyceride accumulation in T0901317- treated HepG2 cells by reducing expression of genes involved in lipogenesis.

### Activation of GPR40 decreases LXR activation-induced lipid accumulation through an AMPK-dependent pathway

**Figure 5:**
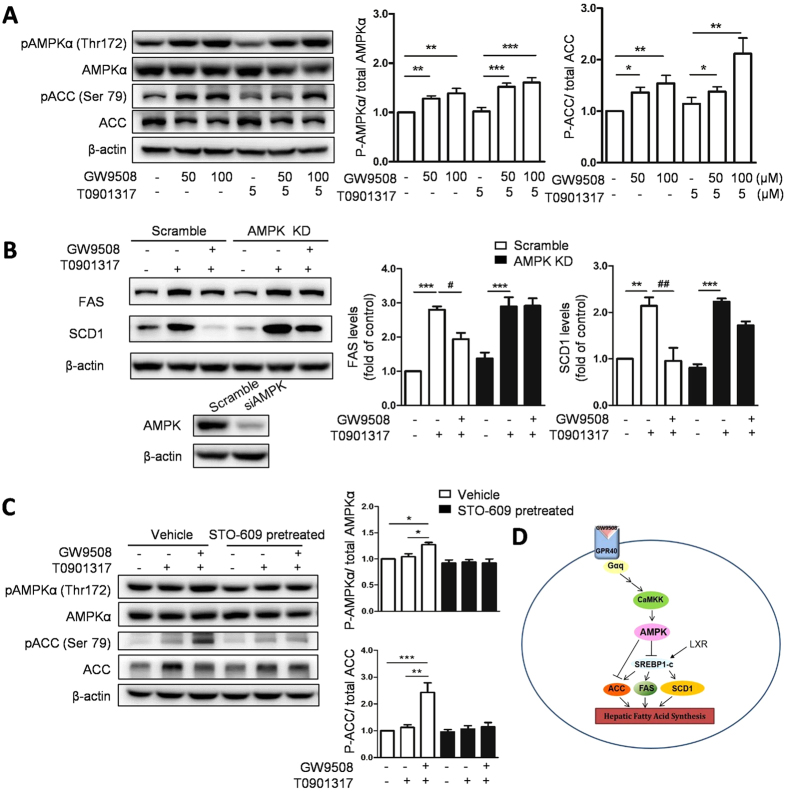
GPR40 reduces LXR activation-induced lipid accumulation by AMPK pathway. (**A**) Western blot analysis and quantitative measurements of phosphorylation of AMPK and ACC in HepG2 cells treated with GW9508 and T0901317. Data are expressed as means ± SEM and obtained from 3 individual experiments. (**B**) Western blot analysis of FAS and SCD1 when AMPK pathway is inhibited. HepG2 cells were transfected with duplexed RNA oligonucleotides of AMPK or scrambled siRNA and pretreated with 100 μM GW9508 for 1 h before 5 μM T0901317 treatment for 24 h. (**C**) Western blot analysis and quantitative measurements of phosphorylation of AMPK and ACC in HepG2 cells pretreated with a CaMKK inhibitor, STO-609, for 1 h prior to the treatment with GW9508 and T0901317. Data are expressed as means ± SEM and obtained from 3 individual experiments. (**D**) The molecular mechanism by which GPR40 participates in LXR-induced hepatic lipid accumulation.

To further clarify the possible mechanism of GPR40 in the regulation of LXR-induced hepatic lipid accumulation, we also examined the phosphorylation level of the AMPK and ACC in HepG2 cells. In accordance with what we have found in mice liver, LXR agonist T0901317 did not affect both phosphorylation of AMPK and ACC in HepG2 cells at a concentration of 5 μM. However, either GW9508 alone or in combination with T0901317 significantly up-regulated the phosphorylation of AMPK and ACC (Fig. 5A).

To further examine if the effect of GPR40 on LXR-induced lipid accumulation was AMPK dependent, we silenced AMPK expression in HepG2 cells using RNA interference and confirmed AMPK expression level by western blot. As expected, in scramble siRNA transfected HepG2 cells, GW9508 treatment abrogated T0901317-induced FAS and SCD1 expression. However, in AMPK-silenced HepG2 cells, GW9508 failed to down-regulate T0901317-induced FAS and SCD1 expression (Fig. 5B). These results suggested that GPR40 ameliorate LXR-induced lipogenesis through an AMPK-dependent pathway.

AMPK Thr172 can be phosphorylated by the Ca^2+^/calmodulin-dependent protein kinase CaMKKβ^22^,^26^,^27^, and intracellular Ca^2+^ concentration can be regulated by GPR40 through phospholipase C as well^28^, suggesting that CaMKK might be the upstream kinase of AMPK when activated by GW9508. To examine this hypothesis, HepG2 cells were pretreated with STO-609, a CaMKK inhibitor, 1 h prior to GW9508 administration, and AMPK activation was detected. Phosphorylation of AMPK and ACC was enhanced by GW9508 treatment, however, this effect was completely diminished by STO-609 pretreatment (Fig. 5C). These results indicated that GPR40 activated AMPK through a CaMKK-dependent manner. Taken together, as shown in Fig. 5D, GPR40 participated in and modulated LXR-mediated hepatic lipid accumulation via activation of AMPK pathway.

## Discussion

GPR40, a sensor of medium- to long- chain fatty acids, has drawn increased attention in recent years for its potential as a novel therapeutic target for metabolic syndrome^29^,^30^. Robust effects of GPR40 agonists on increasing insulin secretion and lowering blood glucose have been shown in rodent model of type 2 diabetes recently. Given the fact that diabetes often correlates with hepatic steatosis, the role of GPR40 in hepatic steatosis is also worth more exploration. In this study, we found for the first time that administration of GW9508 significantly attenuated LXR activation-induced hepatic lipid accumulation by suppressing hepatic lipogenesis in C57BL/6 mice and in HepG2 cells. Moreover, activation of GPR40 with GW9508 attenuated LXR-induced lipid accumulation through stimulating AMPK signaling pathway, in turn inhibiting of LXR activation-stimulated lipogenesis.

It’s believed that increased intracellular cholesterol drives the production of oxysterols which serve as LXR ligands. In hepatocytes, conditions resulting in increased cholesterol enhances synthesis of (24S),25-epoxycholesterol, an oxysterol that specifically binds and activates LXRs^31^. Wouters’s group found that plasma total cholesterol concentration and contents of hepatic total cholesterol and triglyceride significantly increased in C57BL/6 mice only after having been fed a high fat diet with cholesterol (21% milk butter, 0.2% cholesterol) for 2 days^32^ . In the present study, we fed mice with synthetic diet supplemented with 0.2% (w/w) cholesterol for 3 days in order to elevate serum cholesterol level and then provided physiological agonist for activation of LXR in liver. Our study verified that high cholesterol diet feeding for 3 days resulted in increased serum and hepatic total cholesterol level and enhanced the expression of hepatic LXRα, FAS, SCD1 and ACC, and then increased lipid accumulation in the livers of mice. Thus, high cholesterol diet indeed activated hepatic LXR and led to lipid accumulation in livers.

We also found that administration of GW9508 had a beneficial effect on LXR-induced hepatic lipid accumulation by suppressing lipogenesis in mice. According to previous study, administration of GW9508 (25, 50 or 100 mg/kg body weight) could dose-dependently improve glucose intolerance and insulin resistance in high fat diet- induced diabetic mice^16^. Thus, HCD-fed mice were treated with 100 mg/kg GW9508 by gavage once a day in our study. GPR40 activation induced by GW9508 blocked the increased expression of lipogenic genes including FAS, SCD1, and ACC stimulated by LXR. As a consequence, a large amount of small-size lipid droplets deposited in the livers of mice fed a high cholesterol diet were greatly reduced when treated with GW9508 supplementation. However, administration of GW9508 did not change the expression level of hepatic LXRα induced by high cholesterol diet, but directly decreased LXRα-induced expression of lipogenic genes. Additionally, the contents of total and esterified cholesterol in livers were raised by high cholesterol diet feeding, and then were attenuated after being treated by GW9508. The underlying regulation mechanism remain to be elucidated.

Consistent with *in vivo* study, treating HepG2 cells with synthetic LXR agonist T0901317 induced lipid accumulation and the expression of lipogenic genes such as FAS, SCD1, and SREBP1c. However, these effects were largely inhibited by pretreating with GW9508. GPR40 is a Gαq/11- coupled receptor. This kind of G coupled receptor was reported to have the capacity to stimulate the kinase activity of AMPK^33^, an energy sensor that maintains cellular energy homeostasis^34^. A recent study demonstrated that hepatic activation of AMPK by the synthetic polyphenol protected against hepatic steatosis, hyperlipidemia, and accelerated atherosclerosis in diet-induced insulin-resistant LDL receptor deficient mice in part through phosphorylation of SREBP-1c Ser372 and suppression of SREBP-1c- and SREBP-2-dependent lipogenesis^21^. Here, our data showed that GPR40 agonist GW9508 activated AMPK by increasing phosphorylation of AMPK and ACC in HepG2 cells and then inhibited LXR-induced lipogenesis. However, this effect was abolished after AMPK knockdown, suggesting that GPR40 might modulate LXR-induced lipid accumulation by activating the AMPK pathway. Moreover, GPR40 activated AMPK-ACC signaling pathway was CaMKK- dependent. Notably, GW9508 alone could also greatly lower the protein level of SCD1, without affecting its mRNA level and corresponding triglyceride level, suggesting that more signaling pathways may involve in the regulation of lipogenic genes expression by GPR40 activation. For instance, Ou’s study already demonstrated that GW9508 downregulated oleic acid-induced the expression of lipogenesis-related genes through activation of PLC-PKC dependent p38 pathway^17^, posting one more potential signaling pathway downstream of GPR40 activation. However, whether and how GPR40 regulate the protein level of SCD1 in both SREBP-1c-dependent and independent manner still remain to be elucidated.

GPR40 has been considered as a potential target for type 2 diabetes. This receptor contributes significantly to glucose-dependent insulin secretion and thus improves glucose metabolism in both preclinical and clinical studies^35^,^36^. However, the phase III clinical trial of synthetic GPR40 agonist TAK-875 was unfortunately ceased because of the concerns about liver safety, indicating that our understanding of this receptor regarding long-term safety and effects on multiple organs was still very limited. Although GW9508 and TAK-875 share the common pharmacophore, GW9508 is a cell-permeable aminophenylpropanoate and with much smaller molecular weight compared with TAK-875, may have different ways and time for the elimination from the systemic circulation. Since hepatic steatosis is highly linked with insulin resistance and diabetes, research about the role of GPR40 on lipid accumulation in liver has drawn much attention from our group. Although GPR40 agonists are widely applied in the studies of metabolic diseases, the results from animal studies are still controversial and ambiguous. Steneberg *et al*.^37^ found that GPR40 knockout mice were resistant to high-fat diet- induced hyperinslinemia, hyperglycemia, hypertriglyceridemia, and hepatic steatosis. While later studies from Lan *et al*.^38^ showed that GPR40^−/−^ mice had no improvement in adiposity, hyperinsulinemia and lipid accumulations in livers when compared with GPR40^+/+^ mice after high fat diet challenge. When gene manipulating approaches were replaced by chemical agonist, Ou *et al*.^17^ proved a beneficial role of GPR40 agonist GW9508 in hepatic steatosis in high fat diet-fed mice and oleic acid-induced lipid accumulation in HepG2 cells through a p38-dependent pathway. Meanwhile, our study investigated the effect of GPR40 on LXR activation induced- lipid accumulation, and we found that GPR40 agonist GW9508 could also ameliorate LXR activation induced- lipid accumulation by activation of AMPK-ACC pathway, and then inhibiting the expression of hepatic lipogenic genes. It was also worth noting that, in Ou’s study, C57BL/6J mice were fed a high fat diet for 12 weeks, while GW9508 was given for 30 days started at the end of 3 months of high fat diet, giving us a hint that GW9508 was also able to, at least partially, ameliorate hepatic steatosis after lipid accumulation.

In summary, the results in this work provided evidence that GPR40 regulated LXR-induced lipogenesis *in vivo* and *vitro*. Activation of GPR40 by GW9508 inhibited the expression of FAS, SCD1, and ACC induced by LXR and further suppressed lipid accumulation in the livers of mice and HepG2 cells via AMPK pathway. We revealed the significant role of GPR40 and its agonist GW9508 in lipid metabolism, which may provide benefit in the development of new therapeutic approaches for metabolic syndrome in the future.

## Methods

### Materials

Anti-AMPKα, anti-phosphor-AMPKα (Thr172), anti-ACC, anti-phosphor-ACC (Ser 79) and anti-SCD1 antibodies were purchased from Cell Signaling Technology, Inc. Anti-FAS, anti-MTP, anti-PPARα and anti-LXRα antibodies were obtained from Abcam (Cambridge, MA, USA). T0901317 and GW9508 were purchased from Cayman Chemical (Ann Arbor, MI). STO-609 was purchased from Sigma-Aldrich (St. Louis, MO, USA).

### Animals, diets and treatment

C57BL/6 mice were housed in individual cages in a temperature-controlled environment with a 12-h light/dark cycle. The mice were provided water and ad libitum a cereal-based chow diet, unless stated otherwise. In this study, 8-week-old male mice were randomly divided into 3 groups as follows: control (n = 7), C57BL/6 mice were fed a synthetic diet (MD12016, Medicience, Jiangsu, China) for 3 days; high-cholesterol diet (HCD), vehicle treated: C57BL/6 mice (n = 7) were fed a HCD (synthetic diet supplemented with 0.2% (w/w) cholesterol) (MD12016-1, Medicience, Jiangsu, China) for 3 days and given 100 μl vehicle solution (0.4% hydroxypropyl methyl cellulose) by gavage once a day for 3 days; HCD, GW9508 treated: C57BL/6 mice (n = 7) were fed a HCD for 3 days and given GW9508 (100 mg/kg body weight) suspended in 100 μl vehicle solution by gavage once a day for 3 days. Synthetic diet derived 10% calories from fat, 20% calories from protein, and 70% calories from carbohydrate. Mice body weights were recorded, and food intake was calculated as the difference between the food remaining and original food provided, divided by 3 days.

All animal experiments were performed in accordance with recommendations in the National Research Council Guide for Care and Use of Laboratory Animals, with the protocols approved by Animal Care and Use Committee of Beijing Hospital, the Ministry of Health of China.

### Measurement of hepatic and serum lipid concentrations

After a 4 h fast, mice were anaesthetized by intraperitoneal injection with sodium pentobarbital. Blood was collected by heart puncture and liver was removed, weighed, and snap-frozen in liquid nitrogen. The collected blood was centrifuged at 3500 rpm for 10 min at 4 °C, and the serum was analyzed for total and free cholesterol, triglycerides and phospholipids concentrations using the Cholesterol/HP(Roche), the Free Cholesterol C(Wako), Triglycerides/GB kit(Roche) and phospholipids(Wako) enzymatic assay kits, respectively.

For analysis of the liver lipid composition, the lipid extract was separated from liver as described^39^. Lipids were then quantified using the same enzymatic kits described above.

### Oil red O staining

To visualize lipid accumulation in mice liver or in HepG2 cells, oil red O staining was performed according to a previously described method^40^. Briefly, liver cryosections or cells were fixed in calcium formaldehyde solution at room temperature for 15 min, and then dipped in 60% isopropanol for 15 s. The slides were immersed in freshly prepared oil red O working solution for 15 min, and then washed in 60% isopropanol. After washing with PBS, the slides were counterstained with hematoxylin, washed by distilled water, and finally visualized using light microscopy and photographed. For *in vitro* experiments, 1 ml isopropanol was added to each well followed by 10-min shaking at room temperature to elute incorporated oil red O. 200 ul samples were quantified by spectrophotometry at 520 nm.

### Cell culture and treatment

Human HepG2 hepatocytes obtained from Cell Center of Peking Union Medical College were cultured in MEM medium with 10% (v/v) fetal bovine serum, 2 mM L-glutamine, 1 mM sodium pyruvate, and 100 U/mL penicillin, 100 μg/mL streptomycin at 37 °C in a humidified atmosphere containing 5% CO_2_. GW9508 were pretreated at concentrations of 50 or 100 μM for 1 h before incubating with or without T0901317 at a concentration of 5 uM for 24 h (protein extract) or for 48 h (triglyceride and total cholesterol). Each experiment was carried out for at least 3 times separately.

### Measurement of cellular TG and TC content

Cellular lipids were extracted in chloroform/methanol (2/1; v/v), and diluted H_2_SO_4_ was added to the samples, which was vortexed and centrifuged to split the phase. The bottom phase was removed and dried down, then 1% Triton X-100 in CHCl_3_ was added and solvent was evaporated. Lipids were dissolved in deionized water for the determination of TG and TC content using the enzymatic assay kits mentioned above.

### Real-time PCR analysis

Total RNA from cells were isolated using TRIzol reagent (Invitrogen, Carlsbad, CA, USA), then reversely transcribed using Reverse Transcription System (Promega, Madison, WI). Expression of mRNA was quantified by using SYBR Green PCR Master Mix (Takara, Tokyo, Japan) and performed with Bio-Rad real time PCR detection systems according to the manufacturer’s instructions. The following primers were used: SREBP-1c, forward 5′-gga ggg gta ggg cca acg gcc t -3′ and reverse 5′-cat gtc ttc gaa agt gca atc c-3′; FAS, forward 5′- aca ggg aca acc tgg agt tct-3′ and reverse 5′-ctg tgg tcc cac ttg atg agt-3′; SCD-1, forward 5′-tgt tcg ttg cca ctt tct tg-3′ and reverse 5′-gct aat gtt ctt gtc ata agg ac-3′; 18S, forward 5′-taa gtc cct gcc ctt tgt aca ca-3′ and reverse 5′-gat ccg agg gcc tca cta aac-3′. The data were normalized with 18S levels.

### siRNA transfection

HepG2 cells were transfected with specific siRNA oligomers against AMPKα (Santa Cruz) or GPR40 (Dharmacon), using HiPerFect transfection reagent (Qiagen) according to the manufacturer’s instructions. Negative control siRNA oligomers were used as a negative control. After transfection for 24 h, the cells were exposed to GW9508 and T0901317. The silencing of target genes was validated by western blot.

### Western blot analysis

Cell lysates (50 μg of protein) were separated by 8% SDS-PAGE, transferred to PVDF membrane (Milllipore), blocked with 8% nonfat dry milk, and probed with the antibodies at 4 °C overnight. The blots were incubated with HRP-conjugated anti-IgG, followed by detection with ECL (Millipore).

### Statistical analysis

All data were presented as the means ± SEM. The statistical significance of the differences between various treatments was determined by one-way ANOVA (Tukey- Kramer honestly significant difference). *P* value less than 0.05 was considered significant.

## Additional Information

**How to cite this article**: Li, M. *et al*. GPR40 agonist ameliorates liver X receptor-induced lipid accumulation in liver by activating AMPK pathway. *Sci. Rep*. **6**, 25237; doi: 10.1038/srep25237 (2016).

## Figures and Tables

**Table 1:** Serum lipid concentrations.

	**Control**	**0.2% chol**	**0.2% chol GW9508**
BW(g)	22.00 ± 0.36	21.88 ± 0.26	21.36 ± 0.48
Liver /BW ratio	0.052 ± 0.002	0.058 ± 0.002*	0.060 ± 0.001*
Food intake(g/day)	3.57 ± 0.034	3.44 ± 0.135	3.29 ± 0.133
Serum triglyceride(mg/dl)	60.01 ± 2.78	51.29 ± 3.10	49.61 ± 3.76
Serum total cholesterol(mg/dl)	114.41 ± 4.39	148.19 ± 6.33***	153.03 ± 4.74***
Serum free cholesterol(mg/dl)	35.82 ± 1.23	43.55 ± 1.66**	43.14 ± 1.11**
Serum cholesteryl ester(mg/dl)	131.23 ± 6.39	174.73 ± 7.93***	183.53 ± 6.10***
Serum phospholipid(mg/dl)	222.61 ± 4.89	239.23 ± 8.98	239.10 ± 11.95
Serum free fatty acid(mmol/L)	0.405 ± 0.013	0.452 ± 0.020	0.46 ± 0.017

Serum was collected from 4-hour-fasted male mice. Enzymatic kits were used to measure the content of TC, FC, TG, PL, and FFAs in serum. CE content was calculated by multiplying the mass difference between TC and FC by 1.67. Body and liver weight, liver/body weight ratio, and serum lipid content are expressed as mean ± SEM of 6–7 samples. *p < 0.05, **p < 0.01, ***p < 0.001, versus control group.
